# The aesthetic preference for symmetry dissociates from early-emerging attention to symmetry

**DOI:** 10.1038/s41598-018-24558-x

**Published:** 2018-04-19

**Authors:** Yi Huang, Xiaodi Xue, Elizabeth Spelke, Lijie Huang, Wenwen Zheng, Kaiping Peng

**Affiliations:** 1 Department of Psychology, Tsinghua University, Beijing, 100084 P.R. China; 2 Department of Psychology, Harvard University, Cambridge, 02138 USA; 3 Research Center for Brain-inspired Intelligence, Institute of Automation, Chinese Academy of Sciences, Beijing, 100190 P.R. China

## Abstract

Symmetry is a basic geometry property that affects people’s aesthetic experience in common ways across cultures and historical periods, but the origins of the universal preference for symmetrical patterns is not clear. We assessed four-year-old children’s and adults’ reported aesthetic preferences between symmetrical and asymmetrical visual patterns, as well as their spontaneous attentional preferences between the patterns. We found a striking dissociation between these two measures in the children: Children looked longer at the symmetrical patterns, relative to otherwise similar but asymmetrical patterns, but they showed no explicit preference for those patterns. These findings suggest that the human’s aesthetic preferences have high postnatal plasticity, calling into question theories that symmetry is a “core feature” mediating people’s aesthetic experience throughout life. The findings also call into question the assumption, common to many studies of human infants, that attentional choices reflect subjective preferences or values.

## Introduction

People have a universal aesthetic preference for symmetry. Symmetry preferences are manifest in diverse aesthetic forms, from painting and sculpture to architecture and music^1–3^. They influence many aspects of daily life across cultures and historical periods, from purchasing choices^4^ to mate selection^5,6^. As the physicist Herman Wey wrote in his classic book, “beauty is bound up with symmetry”^7^. Is the preference for symmetry a fundamental, natural and innate principle underlying human aesthetic preferences at all ages? Is it reflected in children’s patterns of attention as well as their explicit judgements?

Sensitivity to symmetry has been studied widely in human adults, children and infants with diverse research approaches, from behavioral psychology to neuroscience. Adults detect symmetrical visual displays faster and more accurately than asymmetrical displays^8–12^ and remember them better^13–15^. Their attention to such displays appears to be unaffected by learning^16,17^. With functional magnetic resonance imaging (fMRI), Sasaki and colleagues found that symmetrical visual patterns elicited more activation in the visual cortex^18^. Electroencephalography (EEG) studies have identified a larger sustained posterior negative potential during viewing of symmetrical patterns compared to random ones^19^. At the opposite end of development, 4-month-old human infants take less time to encode visual patterns that are vertically symmetric, relative to asymmetric patterns, providing evidence for early sensitivity to this dimension^20^. These studies do not reveal, however, whether infants or adults have an aesthetic preference for symmetrical patterns.

Symmetry, in some form, plays a role in many art forms^1–3^ and serves as a basic law in graphic design^21^, but its role in guiding the intuitive aesthetic judgments of adults and children is less clear. While Bornstein *et al*. reported more efficient recognition of vertically symmetrical patterns by 4-month-old infants, they also found that only 12-month-old infants show an attentional preference for vertical symmetry compared to horizontal symmetry or asymmetry, as indicated by longer looking times^20^. It is not clear whether this attentional preference has an aesthetic component for infants. Many studies of adults and older children have used explicit judgments to address this issue, and many have focused on the relation between symmetry and facial attractiveness. Adults judge more symmetrical faces to be more attractive^22–25^, and the attractiveness of faces is enhanced by symmetrical decorations. The same effect has been shown for abstract art designs^26^. In one study of children, however, the tendency to judge that symmetrical faces are more attractive developed between 5 and 9 years^27^. Thus, the aesthetic symmetry preference may emerge late and dissociated from the early emerging tendency to attend to symmetrical arrays. It is not clear, however, whether adults’ and older children’s preference for symmetrical faces reflects the emergence of a general aesthetic preference for symmetry or a preference that is specific to the detection of information for a person’s health and vigor: a factor in mate selection^6,24,28,29^. Moreover, it is not clear whether the failure of young children to prefer symmetrical faces stems from limits to their ability to base explicit judgments of any kind on the detection of symmetry.

To address these questions, we tested both adults and four-year-old children’s explicit aesthetic preferences between vertically symmetric and asymmetric visual patterns, while using eye-tracking to record their attention to these patterns. We asked, first, whether young children have an aesthetic preference for symmetrical visual displays over otherwise similar but asymmetrical ones, by describing the displays explicitly as drawings and asking the participants to evaluate their beauty. Then we examined the relationship between these aesthetic judgments and children’s visual exploration of symmetric versus asymmetric patterns. We first presented adults and children with images that were symmetric or asymmetric, and asked them to judge their relative beauty (experiments 1–3). Then we investigated attentional preferences for the same symmetrical patterns in the two populations (experiment 4), to see whether symmetry had comparable effects on aesthetic judgments and on attention.

**Figure 1:**
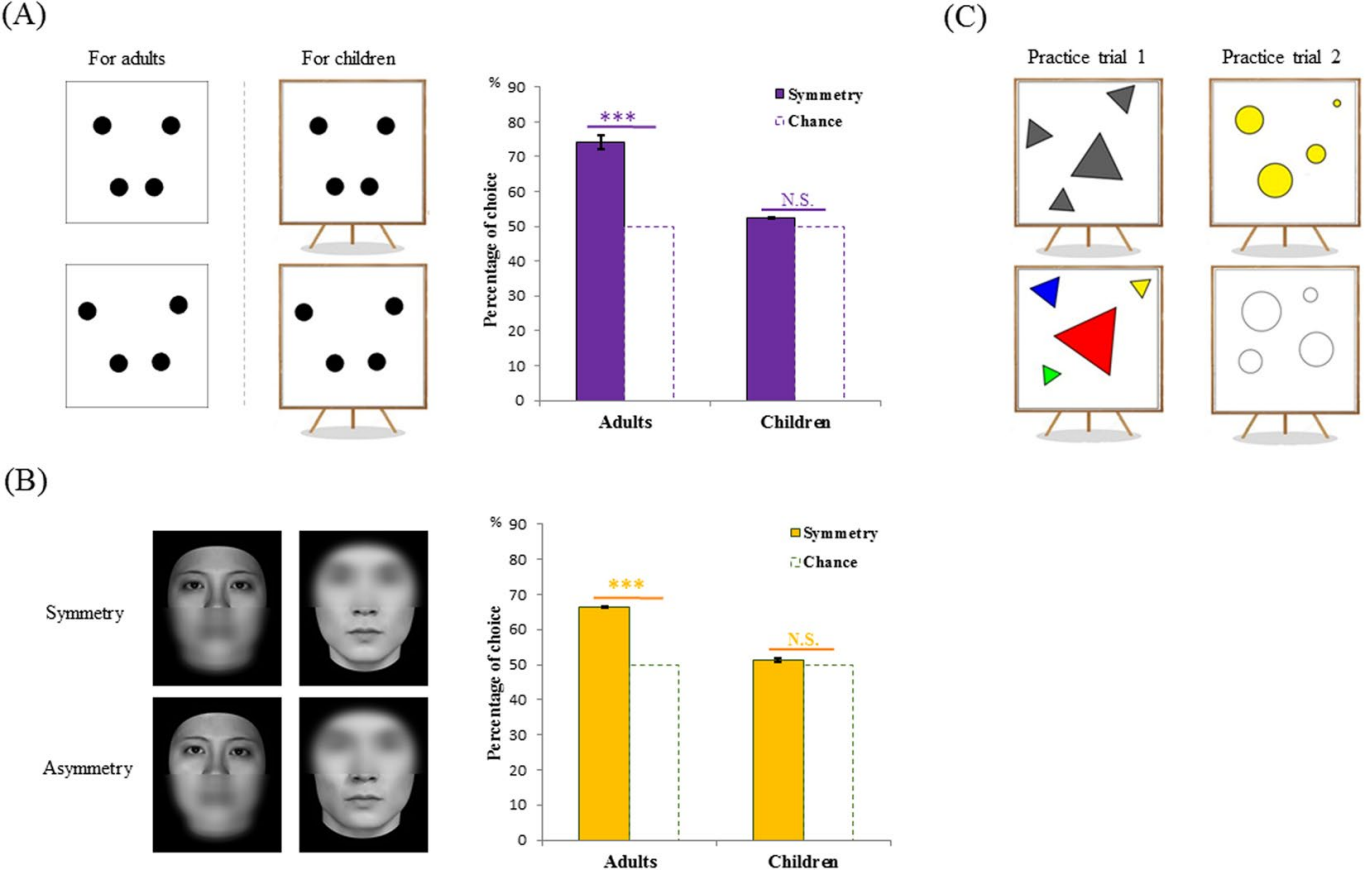
Example displays and findings for experiment 1 (**A**) and 2 (**B**) (the face images were partly blurred only in this figure due to the publication copyright concern), the error bars represent standard errors of the means; (**C**) shows the practice trials given to children at the beginning of experiment 1 and 3. For the children, each dot pattern in the pair was shown with a cartoon teacher (not shown here) presenting the painting on an easel.

In experiment 1, we recruited 101 young children ranging from 3.5 to 5.5 years and 120 adults (*M* = 25.90 years, *SD* = 5.56 years; 55% female); 20 children were eliminated from the final data analysis for failure to pass the practice trials, leaving a sample set of 81 (*M* = 4.36 years, *SD* = 0.8 years; 46% female). Participants were presented with pairs of square pictures, each containing four identical black dots. The dots displayed in one picture were vertically symmetric, whereas those in the other picture were asymmetric (Figs 1A, S2). Other graphic properties were balanced within each pair (see Methods). These simple patterns were chosen in order to minimize the possibility that children would imagine or compare pictures to familiar objects, complicating any interpretation of their aesthetic judgements.

For the adults, the testing was programed on a smart phone, which displayed each pair of drawings and instructed participants to choose which member of pairs was more beautiful or more attractive by pressing the button next to the picture. Participants were told to rely on their first impression or intuition without too much thought or analysis. A preliminary experiment, performed on computer and with reaction time recorded, showed normal adult participants could complete a choice within 1.41 seconds on average (*N* = 34, *SD* = 0.66 s). In order to increase children’s interest of engagement and sustain their attention, the program was presented to children as a game played with the instructor (experimenter or parents) and the number of trials was reduced to 10 (randomly chosen from the original 20 items), but the study rationale stayed the same. In each trial of the game, the instructor told the children that, teacher Xiao Ming and teacher Xiao Mei would present their own painting works (Fig. 1A), and the job of the child was to decide which teacher’s painting was more beautiful by pointing it out with their finger. The experimenter recorded the child’s answer. Before the main test, we added two pairs of practice trials. In the practice trials, the key difference between the two paired pictures was that one was brightly colored whereas the other was black and white (Fig. 1C). Because children are known to prefer brightly colored objects^30^, these practice trials both served as a warm up and probed whether children understood the task.

The percentage of choices of the symmetrical pattern was calculated and compared with the chance level of 50%. We found that adults significantly choose the symmetrical pictures as more beautiful (*M* = 74.17%, *SD* = 21.44%, *t* (119) = 12.35, *p* < 0.001, *d* = 1.13, *95% CI* [20.29, 28.04]). In contrast, children did not show this preference: they chose symmetrical pictures no more than asymmetrical ones (*M* = 52.35%, *SD* = 17.05%, *t* (80) = 1.24, *p* = 0.22, *d* = 0.13, *95% CI* [−1.424, 6.12]) (Fig. 1A). Thus, the symmetry preference was not manifested in this young age.

Why did children not show a symmetry preference? Is that the task was not valid for them. To address this possibility, we analyzed children’s performance on the practice trials which contained pictures either with bright colors or in black and white. We found that 80.2% of the children (81 out of 101, binomial test: *p* < 0.001) chose the colored pictures as more beautiful on both practice trials, ruling out this concern.

Though we used simple dot patterns to avoid the confounding of prior visual experience, and we controlled other visual features such as balance and crowding scores for the picture pairs, a second possible reason for children’s failure to respond to symmetry stemmed from the competitive effect of dot proximity^31,32^ (we thank an anonymous reviewer for suggesting this interpretation). To test this possibility, we calculated the correlation between the crowding score of each picture (see Table S1) and participants’ preference choices across the picture pairs. No significant correlation was found for children (symmetry: *r* = −0.26, *p* = 0.47; asymmetry: *r* = −0.03, *p* = 0.93) or adults (symmetry: *r* = −0.22, *p* = 0.35; asymmetry: *r* = −0.05, *p* = 0.83). Thus, the difference between children’s and adults’ subjective aesthetic preferences was not due to the influence of spatial proximity on children’s sensitivity to symmetry.

A third factor that might explain the difference between the preference choices of children and adults was that the plain dot patterns were too abstract or unfamiliar to the young children. To eliminate this confounding factor, we conducted experiment 2, which presented children and adults with pictures of human faces. A new group of 33 children (*M* = 4.41 years, *SD* = 0.70 years; 52% female) and 91 adults (*M* = 29.01 years, *SD* = 6.16 years; 65% female) viewed pairs of photographs of young males or females with hair and clothing covered or removed. Each pair contained one original photo and another one adjusted to achieve perfect symmetry (Fig. 1B, see more image processing details in Materials and Methods of Supplementary Materials). In accord with the findings of the experiment using the dot patterns, the children did not choose the more symmetrical faces as more beautiful (*M* = 51.21%, *SD* = 14.74%, *t* (32) = 0.47, *p* = 0.64, *d* = 0.08, *95% CI* [−4.01, 6.44]), although the adults did (*M* = 66.43%, *SD* = 14.09%, *t* (90) = 11.12, *p* < 0.001, *d* = 1.17, *95% CI* [13.49, 19.36]) (Fig. 1B). This result ruled out the possibility that children failed to show aesthetic symmetry preference in experiment 1 because the dot patterns were too abstract. Together, experiments 1 and 2 provided evidence for an aesthetic symmetry preference in adults but not children, both for the abstract well-controlled dot patterns and for highly familiar and meaningful human faces.

It remains possible, however, that the greater number of trials given to adults relative to children accounted for their differing findings. Experiment 3 tested that possibility. A new group of children (*N* = 44, *M* = 4.35 years, *SD* = 0.49 years; 43% female) was recruited in kindergarten with the same experimental procedure and stimuli type as in the children’s version of experiment 1, but with the stimuli of original 20 trials given to adults. The result was compared with the result of experiment 1. If the differing performance of children and adults stemmed from the greater number of trials given to adults, then the children in the present experiment should show the same aesthetic preference for the symmetrical patterns as did the adults. On the other hand, if the differing performance of children and adults is robust and generalizable, then the children in the present experiment should perform like those in experiment 1, despite the differing test context (school versus home) and the doubling of the number of trials. The results replicated the findings of experiments 1 and 2. Four-year-old children did not show any aesthetic preference for the symmetrical patterns (*M* = 51.02%, *SD* = 12.60%, *t* (43) = 0.54, *p* = 0.59, *d* = 0.08, *95% CI* [−2.81, 4.85]), and did not differ from the children in experiment 1 (*t* (123) = −0.45, *p* = 0.65, *d* = 0.09, *95% CI* [−7.12, 4.47]). These findings both replicate and extend the findings of Vingilis-Jaremko and Maurer^27^. They show that young children fail to use symmetry either in judging the attractiveness of human faces or in judging the aesthetic quality of drawings of abstract patterns.

Next, we tested children’s attentional preference towards the visual patterns to test two further possible reasons for children’s failure to respond to symmetry. First, symmetry may not be detectable in the present displays, because the differences between the symmetrical and asymmetrical faces were subtle, and the symmetrical and asymmetrical dot patterns were unfamiliar and atypical examples of paintings. Thus, young children may have an aesthetic preference for symmetrical patterns that failed to appear, because the symmetry was not perceived. Second, young children may notice this special visual structure, but their aesthetic system may not treat it as a pleasurable pattern. To distinguish these possibilities, in experiment 4, we tested whether 4-year-old children, like infants, look longer at the symmetrical patterns used in experiment 3. If children fail to detect the symmetrical patterns, then indeed they may have the same aesthetic preferences as adults, but lower perceptual sensitivity to symmetry. If children successfully detect the symmetrical patterns by the attention measure, then the apparent lack of an aesthetic preference for symmetry would appear to stem from the immaturity of their aesthetic judgments.

Another group of 4-year-old children (*N* = 43; *M = *4.51 years, *SD* = 0.52 years; 44% female) was recruited in kindergarten for the eye-tracking study in experiment 4; meanwhile, another group of adults (*N* = 51; *M* = 24.72 years, *SD* = 5.19 years; 59% female) was recruited on the university campus. The eye tracking test was performed in a quiet room with the SensoMotoric Instruments Red 5 system. Same dot pattern pairs used in experiment 3 were presented on the center of monitor screen vertically as they were presented in experiment 3. Twenty trials and 3 practice trials (2 were same as for practice in the experiment 1 and 3, plus with another practice trial using mirror reflected cartoon characters) were included both for children and adults. In order to avoid effects of attention to irrelevant features of the displays, the cartoon teacher characters and easels used on the pictures in experiment 1 and 3 were removed for the eye tracking experiment. The symmetrical picture were randomly shown on the bottom or the top, and the locations were balanced across 20 trials. Each stimulus was presented 4000 ms for each trial, the interval between trials was 3000 ms, following the untimed practice trials. For the data analysis, the whole screen was divided into two regions of interest (symmetrical vs. asymmetrical) along the horizontal midline of the screen. Then the fixation frequencies and durations were calculated for each region of interest.

**Figure 2:**
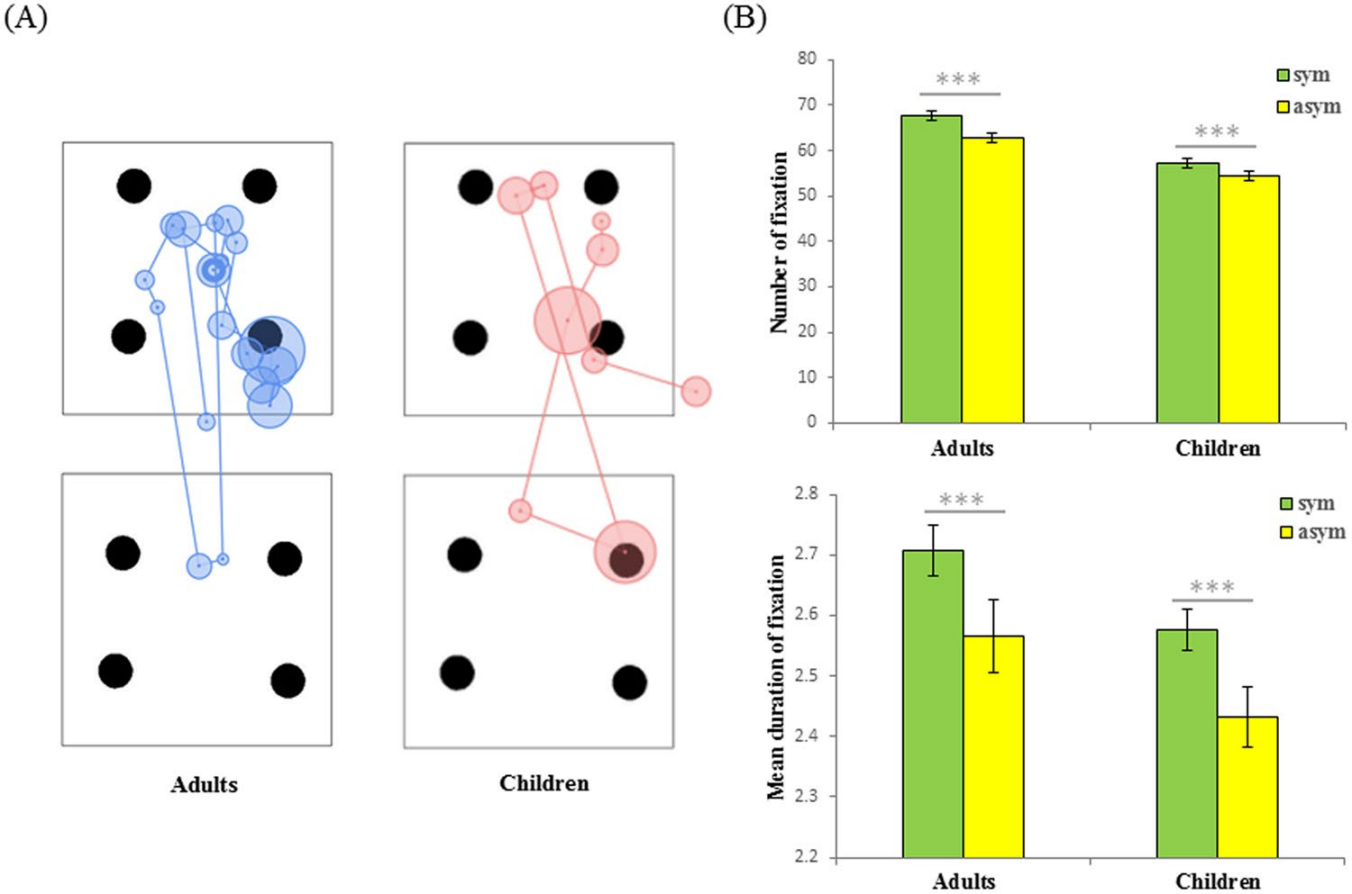
Example eye-movement paths (**A**) and the statistic results (**B**) for the adults and 4-year-old children in experiment 4, the error bars represent standard errors of the means.

We found that children and adults differed on the aesthetic symmetry preference, but their behavior on the visual attention test was similar: both children and adults had more fixations on the symmetrical patterns compared with the asymmetrical ones (children: *t* (42) = 3.04, *p* = 0.004, *d* = 0.46, *95% CI* [0.99, 4.92]; adults: *t* (50) = 8.06, *p* < 0.001, *d* = 1.13, *95% CI* [3.79, 6.31]), as well as longer fixation durations for the symmetrical patterns compared with the asymmetrical ones (children: *t* (42) = 2.98, *p* = 0.005, *d* = 0.45, *95% CI* [0.05, 0.24]; adults: *t* (50) = 4.35, *p* < 0.001, *d* = 0.61, *95% CI* [0.08, 0.21]) (Fig. 2). These findings accord with the findings of the experiments with infants^20^, showing looking preferences for vertically symmetrical patterns. Together with the findings of experiments 1–3, this experiment showed that a subjective aesthetic preference for symmetry does not emerge together with the perception and attention to symmetry. In young children, aesthetic preferences dissociate from attentional choices, at least for the present arrays, providing evidence that the aesthetic preference for symmetry develops later than the perceptual sensitivity to symmetry.

From philosophers, mathematicians, physicists, artists to psychologists and neuroscientists, many scholars have been fascinated by symmetry and have pondered the nature of its effects on us. In the current experiments, we found that people’s judgments of the aesthetic appeal of a work of art was affected by symmetry for adults but not at the age of four. Four-year-old children were sensitive to symmetry in the present patterns, but this sensitivity did not guide their aesthetic judgments, as it did for adults. To our knowledge, our findings provide the first direct evidence for a dissociation between attention to symmetry and aesthetic preferences for symmetry at this young age. More generally, these results suggest that the aesthetic “core feature” of symmetry, which is often considered as a basic, foundational rule of beauty, emerges late in human development.

The present findings cast doubt on the processing fluency theory of aesthetic judgment, at least for young children, whereby aesthetic pleasure is promoted by the redundancy of information in symmetrical displays^33^. Although children’s visual acuity is not fully mature at 4 years of age^34,35^, and signal detection efficiency increases from ages of 5 to 9^36^, 12-month-old infants detect and attend to vertical symmetry in patterns, and 4-month-old infants show shorter inhibition time towards vertical symmetry, suggesting an early ability to use the redundancy of symmetrical patterns to increase children’s processing efficiency^20^. In our experiments, 4-year-old children showed these effects when presented with the same patterns that elicited no aesthetic preferences. Why does the aesthetic preference for symmetry develop so late, when children detect symmetry even as infants?

To approach this question, the roles of phylogeny and of visual experience both should be considered. For facial attractiveness, symmetry implies health and therefore good mate quality^6,24,28,29,37^. For young children, however, mate quality likely has no meaning or rewarding value. If this is true, then adolescents after puberty should pay more attention to symmetry related facial attractiveness, and this indeed has been confirmed by studies^38,39^. Thus, phylogenically, the development of aesthetic system may be more complex than the development of perceptual system, as well as the development of their connections.

Another factor that may influence the late development of aesthetic preference system is the accumulation of postnatal experience. The mere exposure theory of human preferences proposes that people prefer objects to which they have had greater exposure, and this effect can be produced in very short time windows of seconds for adults^40,41^. Might the aesthetic symmetry preference arise from adults’ greater exposure to symmetrical objects in daily life? In a relative longer time window, studies had found children’s aesthetic judgements are affected by mere exposure too. For example, adapting 8-year-old children and adults to faces with either compressed or expanded features let them to increase their attractiveness ratings of faces in the distorted direction, compared to before adaptation^42^. And the experience of interaction with peers affects 3-year-old children’s judgement of facial attractiveness related to feature proportion^43^. However, the interaction experience of faces was directly given in these studies, which may have increased the salience of the faces for children. Perceptually, salience had been found to influence on infants’ visual attention^44–46^; and children’s sensitivity to the global structure present in dot patterns, moving dots, or biological motion continues to improve until the 9–12 years of age^47,48^. Thus, children have less experience with symmetrical objects and the symmetrical patterns they do experience are combined with other figural properties. Plus, adults have more chances to gain symmetry-related experience by studying art and design. Lots of evidence shows that aesthetic expertise affects people’s level of aesthetic appreciation^49,50^. Future studies could test whether giving children direct experience of symmetry prompts emergence of an aesthetic symmetry preference.

The methods of neuroscience provide another approach that may help to untangle the relation between attention to symmetry and aesthetic symmetry preferences in human development. By means of EEG, Jacobsen *et al*. reported that perceptual judgments of symmetry are performed faster than evaluative appreciation of their beauty for adult participants^51^, indicating the separable processes of perception process and aesthetic judgement in adulthood. In the aesthetic triad theory proposed by Chatterjee *et al*., based on the previous neuroaesthetic studies, aesthetic experience is regarded as an emergent state, arising from interactions between sensory–motor, emotion–valuation, and meaning–knowledge neural systems^52^. The separation and interaction between these systems, as well as their relation with other neural systems underlying emotion and reward, during development is worthy of further investigation.

Our finding about the separation between attentional choices and aesthetic preference in young children also calls into question the assumption, common to many studies of human infants, that attentional choices reflect subjective preferences based on abstract qualities such as beauty or moral value^53,54^. It is possible that the attentional preferences may provide the preliminary basis for the development of children’s aesthetic preferences, however, whether these two can be equally treated as reflecting each other, especially in the very young stage, would need to be considered more carefully. Our finding call for caution in interpreting early developing looking patterns in infants. Such patterns may or may not indicate preferences for attended object^32^. Our findings also suggested caution in interpreting methods, such as ‘oculomotor genetic algorithms’, which equate fixation frequency and duration with aesthetic preference^55,56^.

Some limitations in the current study should be noted and addressed by future research. First, though visual features other than symmetry were controlled in the present picture pairs, it was hard to completely avoid the difficulty in measuring preference for one visual dimension at a time^57^. For example, a symmetric dot pattern may contain implicit horizontal parallel lines whereas the asymmetry pattern does not, and the “oblique effect” suggest a possible aesthetic advantage of horizontal/vertical parallels^58,59^. Second, the interaction between different features, such as spatial proximity, the sensitivity of which develops during childhood, may cause difference in pattern salience to adults versus children; the salience of symmetry, moreover correlates with aesthetic experience^60^. One way we propose to overcome these problems is to perform the tests with more variation of the symmetry/asymmetry, so that the findings can be convergently manifested even if each variation has some hard-to-avoid defects. Finally, we cannot determine whether differences in meta-cognitive processes between children and adults contributed to the current findings. For example, adults may because quickly aware of the experimental task focused on symmetry versus asymmetry, and this explicit awareness may promote them to make more consistent “liking” choices. Some of our unpublished data showed that when explaining what the concept of symmetry is to 4-year-old children (*N* = 33; *M* = 4.15 years, *SD* = 0.63 years; 60.6% female), they can readily discriminate symmetric from asymmetric dot patterns (*M* = 85%, *SD* = 17.90%, *t* (32) = 11.23, *p* < 0.001, *d* = 0.08, *95% CI* [28.65, 41.35]), but we are not sure whether or not these properties are automatically salient to them. As the practice trials used a different visual feature (color) for the children, it is possible that these trials interfered with the subsequent symmetry/asymmetry trials. These factors should be further considered in future studies.

## Methods

### Materials and Procedure

#### Dot Pattern and Face test

For the dot pattern test, 20 pairs of square-shaped pictures that each contains 4 identical black dots generated by Matlab customized code were used as stimuli. The dot pattern was vertically symmetric in one member of each pair, but asymmetric in another (Figs 1A, S2). Other graphic properties (e.g. balance of the pattern, average dot-to-center distance/crowding degree) were balanced between pictures within each pair (see more details in Materials and Methods, Figure S1 and Table S1 of Supplementary Materials)^61^. These simple dot patterns were chosen in order to minimize the possibility that children would imagine or compare pictures to familiar objects, such as flowers or butterflies etc., complicating any interpretation of their aesthetic judgements.

For the face test, 20 pairs of photographs of young males or females in their front side were used, and all their un-facial information, like hair and personal wearing were covered or removed. Each pair contained one original photo and another one adjusted to achieve perfect symmetry (Fig. 1B, see more image processing details in Materials and Methods of Supplementary Materials).

The experiment program was delivered as an html 5 page compatible on smart phones. Participants could get and play the program through Wechat, which is a most popular social network application in China. The adults and parents that participating the study were completely voluntary and were given a small monetary incentive automatically assigned by the program (2–5 Yuan RMB). The program also collected consent before the formal test.

For the adult testing, participants were instructed to choose which one of two pictures was more beautiful or more attractive to them by pressing the button next to the picture. No time limit was imposed, but participants were told to rely on their first impression or intuition without too much thought or analysis. Twenty pairs of pictures with dot patterns or faces were used for 20 trials (Fig. S2). After the main experiment, participants completed a sheet giving personal information including age, gender, date of birth, education level and academic major.

For the children’s version of dot pattern test, in order to increase children’s interest of engagement and sustain their attention, the program was presented to children as a game played with the instructor (experimenter or parents) and the number of trials was reduced to 10 (randomly chosen from the original 20 items), but the study rationale stayed the same. In each trial of the game, the instructor told the children that, teacher Xiao Ming and teacher Xiao Mei would present their own painting works (Fig. 1A), and the job of the child was to decide which teacher’s painting was more beautiful by pointing it out with their finger. The experimenter recorded the child’s answer. Before the main test, we added two pairs of practice trials. In the practice trials, the key difference between the two paired pictures was that one was brightly colored whereas the other was black and white (Fig. 1C). Because children are known to prefer bright colored objects (Suchman & Trabasso, 1966), these practice trials both served as a warm up and probed whether children understood the task. The sheet of personal information about the child and the parents including age, gender, date of birth, education level and major was filled by the instructor at the end of the game. For the face test for children, the game setting was let them judge which photograph in each pair was taken more beautifully and no cartoon character was shown for this test.

All the experimental protocols were approved and performed under the regulation of Institutional Review Board (IRB) for human research of Department of Psychology in Tsinghua University. And the informed consent was obtained from each participant.

#### Eye Tracking Test

The eye tracking test was performed with the SensoMotoric Instruments Red 5 system setting in a quiet room, with the sample frequency of 120 HZ, the presentation screen resolution of 1680 × 1050 pixels, and the distance between screen and head tray of 65 cm. Five points calibration was used for both children and adults. The dot pattern pictures in each pair were presented vertically on the center of screen as they were presented on the smart phone in experiment 1 and 3. The size of each picture was 320 × 320 pixels, and the distance between the two pictures in each pair was 80 pixels. Twenty trials and 3 practice trials (2 were same with practice in the experiment 1 and 3, plus with another practice trial using mirror reflected cartoon characters) were included both for children and adults. The symmetrical or asymmetrical picture was randomly shown on the bottom or on the top, and the locations were balanced across 20 trials. Each stimulus was presented 4000 ms for each trial, and the interval between trials was 3000 ms. No time limit for the practice trial until the participant made the choice. For the data analysis, the whole screen was divided into two regions of interest (symmetrical versus asymmetrical) along the horizontal midline of the screen. Then the fixation frequencies and durations were calculated for each region of interest. All the experimental protocols were approved and performed under the regulation of IRB for human research of Department of Psychology in Tsinghua University. And the informed consent was obtained from each participant.

## Electronic supplementary material

Supplementary information
