# Supplementary material for: The aesthetic preference for symmetry dissociates from early-emerging attention to symmetry
Source: Sci Rep. 2018 Apr 19;8:6263. doi: 10.1038/s41598-018-24558-x (PMC5908848; doi:10.1038/s41598-018-24558-x)
Supplement: Supplementary file 1 — Supplementary information [file 41598_2018_24558_MOESM1_ESM.doc]

**Title:** The aesthetic preference for symmetry dissociates from early-emerging attention to symmetry

**Authors:** Yi Huang^1^*, Xiaodi Xue^1^, Elizabeth Spelke^2^, Lijie Huang^3^, Wenwen Zheng^1^, Kaiping Peng^1^*

**Affiliations:**

^1^ Department of Psychology, Tsinghua University, Beijing, 100084, PR China.

^2^ Department of Psychology, Harvard University, Cambridge, 02138, USA.

^3^ Research Center for Brain-inspired Intelligence, Institute of Automation, Chinese Academy of Sciences, Beijing, 100190, PR China .

***Correspondences:**

Yi Huang, Department of Psychology, Tsinghua University, Beijing, 100084, PR China; +86 13811849363; yhuang83@gmail.com

Kaiping Peng, Department of Psychology, Tsinghua University, Beijing, 100084, PR China; +86 010- 62792784; pengkp@mail.tsinghua.edu.cn

Supplementary Information:

Materials and Methods

Figures S1-S2

Tables S1

**Materials and Methods**

**Dot pattern creation in experiment 1 and 3.** The stimuli pictures were created on computer by customized Matlab code. For the symmetrical ones, firstly, a 400 X 400 white background image with black outline was divided as two half parts by a virtual vertical midline. Secondly, on the left half, two black dots whose diameter was 20 pixels were drawn at random locations without any overlap. Then the mirror image of the dots along the vertical midline was projected to the right half. While for the asymmetry picture, all of the four dots were randomly located on the white page.

For every unique pair of pictures, the balance and crowding scores of the two pictures were strictly balanced. *The balance score* was calculated with the method of Wilson and Chatterjee [^1^](#_ENREF_1). In specific, the white 400×400 pixels square background image was firstly divided into four pairs of equal parts along the two principal axes: the vertical axis, the horizontal axis; and two diagonal axes, bottom left to top right, and bottom right to top left axis (see Figure S1A). Secondly, four additional dividing for creating “inner-outer” divisions of the areas were created by three vertical or horizontal dividing lines (Figure S1B). Therefore, eight pairs of divided areas along different dividing lines were created. Next, for each pair of the divided areas, the measures of balance score was calculated as follows: (|area 1 – area 2|) / (area 1 + area 2) × 100. And the final balance score of a picture was the average of eight measures. And the *crowding score* was calculated as the area of the quadrilateral consisted by the four line segments whose endpoints were the centers of the dots. In each pair of pictures, the balanced scores were exactly the same; and the crowding scores (Table S1.) between the symmetrical pictures and asymmetrical pictures had no significant difference (*t (19)* =0.15, *p* = 0.88).

**Face stimuli preparation for experiment 2.** Sixty candidate pictures of young male or female in their front side were photoed, and all un-facial information, like hair, personal wearing were covered and removed. Then each face picture was cut into two half parts along the vertical axis; then one half part (left or right) was taken out and combined together with its own mirror reflected copy to become a new face that has perfect symmetry property. Then each new face were paired with the original one to become the stimuli of a trial. Because not every mirror reflected human face would look natural and had more subjective beauty, we recruited an extra group of adults subject (*N* = 203; *mean age* = 29.66, *SD* = 6.03; 65% female) to choose which one of each paired face was more beautiful and then 20 pairs which had natural and more beautiful symmetrical faces were selected for formal test both on children and adults.

**Figures S1-S2**


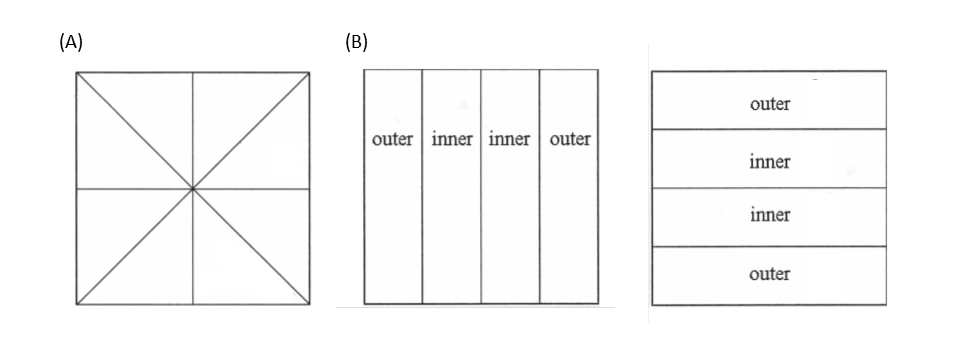


**Figures S1.** The area dividing method when calculating the balance score for each stimulus of dot pattern pictures.


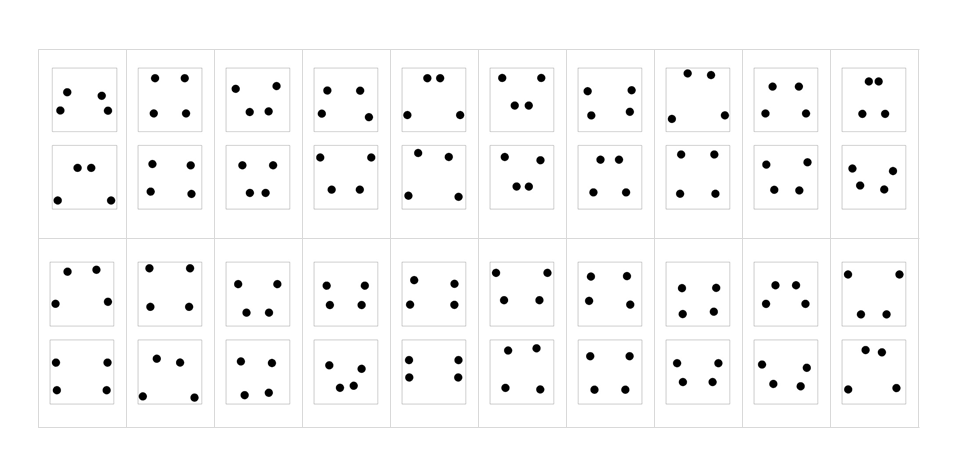


**Figures S2.** All 20 pairs of the dot patterns used as stimuli for the experiments.

**Table S1**

**Table S1.** The list of balance score and crowding score for each pair of dot pattern pictures.

| **Picture Pairs** | **Balance Score** | | **Crowding Score (CS)** | | **T-test on CS** | |  |
| --- | --- | --- | --- | --- | --- | --- | --- |
|  | **Symmetry** | **Asymmetry** | **Symmetry** | **Asymmetry** | **t** | **p** |  |
| P1 | 0.38 | 0.38 | 145.8093 | 137.2929 | 0.15 | 0.88 |  |
| P2 | 0.23 | 0.23 | 147.9192 | 151.813 |  | |  |
| P4 | 0.22 | 0.22 | 159.4861 | 147.2683 |  | |  |
| P5 | 0.33 | 0.33 | 165.8023 | 182.8574 |  | |  |
| P6 | 0.22 | 0.22 | 119.8611 | 115.729 |  | |  |
| P7 | 0.33 | 0.33 | 131.6783 | 148.6702 |  | |  |
| P8 | 0.26 | 0.26 | 163.5793 | 187.2061 |  | |  |
| P9 | 0.35 | 0.35 | 134.6855 | 133.6456 |  | |  |
| P10 | 0.42 | 0.42 | 115.9251 | 117.6237 |  | |  |
| P11 | 0.22 | 0.22 | 181.8355 | 171.7687 |  | |  |
| P12 | 0.32 | 0.32 | 174.683 | 162.7777 |  | |  |
| P13 | 0.45 | 0.45 | 136.1582 | 135.1301 |  | |  |
| P14 | 0.42 | 0.42 | 126.0018 | 132.7498 |  | |  |
| P15 | 0.45 | 0.45 | 125.3076 | 103.8278 |  | |  |
| P16 | 0.35 | 0.35 | 164.0661 | 150.3302 |  | |  |
| P17 | 0.35 | 0.35 | 175.4891 | 161.0369 |  | |  |
| P18 | 0.3 | 0.3 | 151.7787 | 148.7428 |  | |  |
| P19 | 0.43 | 0.43 | 110.942 | 131.3373 |  | |  |
| P20 | 0.24 | 0.24 | 162.3128 | 158.1048 |  | |  |

**References**

1 Wilson, A. & Chatterjee, A. The assessment of preference for balance: Introducing a new test. *Empirical Studies of the Arts* **23**, 165-180 (2005).
